# Renal cell carcinoma alters endothelial receptor expression responsible for leukocyte adhesion

**DOI:** 10.18632/oncotarget.7804

**Published:** 2016-03-01

**Authors:** Eva Juengel, Geraldine Krueger, Jochen Rutz, Karen Nelson, Isabella Werner, Borna Relja, Barbara Seliger, Beate Fisslthaler, Ingrid Fleming, Igor Tsaur, Axel Haferkamp, Roman A. Blaheta

**Affiliations:** ^1^ Department of Urology, Goethe-University Hospital, Frankfurt am Main, Germany; ^2^ Department of Vascular and Endovascular Surgery, Goethe-University Hospital, Frankfurt am Main, Germany; ^3^ Department of Thoracic, Cardiac and Vascular Surgery, Goethe-University Hospital, Frankfurt am Main, Germany; ^4^ Department of Trauma, Hand and Reconstructive Surgery, Goethe-University Hospital, Frankfurt am Main, Germany; ^5^ Institute for Medical Immunology, Medical Faculty, Martin Luther University, Halle, Germany; ^6^ Institute for Vascular Signalling, Centre for Molecular Medicine, Goethe-University, Frankfurt am Main, Germany

**Keywords:** adhesion, endothelial receptor expression, leukocytes, renal cell carcinoma, tumor immune escape

## Abstract

Renal cell carcinoma (RCC) escapes immune recognition. To elaborate the escape strategy the influence of RCC cells on endothelial receptor expression and endothelial leukocyte adhesion was evaluated. Human umbilical vein endothelial cells (HUVEC) were co-cultured with the RCC cell line, Caki-1, with and without tumor necrosis factor (TNF)-alpha. Intercellular cell adhesion molecule-1 (ICAM-1), vascular cell adhesion molecule-1 (VCAM-1), endothelial (E)-selectin, standard and variants (V) of CD44 were then analysed in HUVEC, using flow cytometry and Western blot analysis. To determine which components are responsible for HUVEC-Caki-1 interaction causing receptor alteration, Caki-1 membrane fragments versus cell culture supernatant were applied to HUVECS. Adhesion of peripheral blood lymphocytes (PBL) and polymorphonuclear neutrophils (PMN) to endothelium was evaluated by co-culture adhesion assays. Relevance of endothelial receptor expression for adhesion to endothelium was determined by receptor blockage. Co-culture of RCC and HUVECs resulted in a significant increase in endothelial ICAM-1, VCAM-1, E-selectin, CD44 V3 and V7 expression. Previous stimulation of HUVECs with TNF-alpha and co-cultivation with Caki-1 resulted in further elevation of endothelial CD44 V3 and V7 expression, whereas ICAM-1, VCAM-1 and E-selectin expression were significantly diminished. Since Caki-1 membrane fragments also caused these alterations, but cell culture supernatant did not, cell-cell contact may be responsible for this process. Blocking ICAM-1, VCAM-1, E-selectin or CD44 with respective antibodies led to a significant decrease in PBL and PMN adhesion to endothelium. Thus, exposing HUVEC to Caki-1 results in significant alteration of endothelial receptor expression and subsequent endothelial attachment of PBL and PMN.

## INTRODUCTION

Renal cell carcinoma (RCC) is the most common renal tumor, accounting for 3% of all malignant tumors in adults and the incidence is increasing [1]. Approximately a third of these patients already have metastases at diagnosis, while up to 30% of patients develop metastases during therapy. Once metastasized, the prognosis of RCC patients is poor. RCC is considered an immunogenic tumor and angiogenesis dependent [2]. During the last three decades RCC patients have received immunotherapy based on interleukin-2 (IL-2) or interferon-alpha (IFN-α). But these cytokines have only been marginally successful, with a response rate between 10% and 15 % and at cost of severe side effects. Better understanding of the molecular mechanisms involved in RCC has resulted in the development of different targeted therapies, which have significantly improved therapeutic outcome. However, the prognosis for patients with RCC still remains poor, with 5-year survival lying between 5%-12% [3, 4].

Immune surveillance evasion is one factor involved in RCC progression [2] and tumor necrosis factor (TNF)-alpha is an early indicator of malignancy [5]. In the presence of TNF-alpha, particularly the endothelial adhesion receptors intercellular cell-adhesion molecule-1 (ICAM-1), vascular cell adhesion molecule-1 (VCAM-1), endothelial (E)-selectin and CD44 variants are elevated [6–8]. ICAM-1, VCAM-1, E-selectin and CD44 variants are highly expressed in a number of malignancies and mediate leukocyte binding to the vascular endothelium, thereby facilitating endothelial transmigration [6–9]. Especially peripheral blood lymphocytes (PBL) and polymorphonuclear neutrophils (PMN) bind to ICAM-1, VCAM-1, E-selectin and CD44 variants [8, 10–12]. When and where leukocyte extravasation takes place is determined by adhesion receptors on endothelial blood vessel cells. Interaction between tumor, endothelium and immune cells appears pivotal to the RCC immune escape during hematogenous metastasis [13]. Therefore, the influence of RCC cells on endothelial adhesion receptors, which regulate leukocyte extravasation, was investigated.

## RESULTS

### Endothelial adhesion receptor stimulation by cytokines

**Figure 1:**
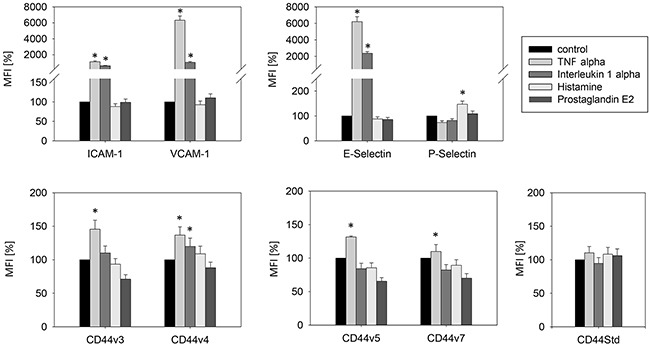
Stimulation of endothelial relevant immune response receptors in HUVEC by TNF-alpha (500 U/ml, 16h), histamine (100 μM/ml, 10 min), interleukin 1 alpha (500 U/ml, 16h) and prostaglandin E2 (10 μM/ml, 10 min) MFI = mean relative fluorescence intensity. *indicates significant difference to untreated controls. n=5.

Optimal stimulation of endothelial relevant receptors to illicit an immune response on the surface of HUVEC by the cytokines, TNF-alpha, IL-1-alpha, histamine and prostaglandin E2 was evaluated by reaction kinetics. TNF-alpha induced significant increases in HUVEC surface expression of ICAM-1, VCAM-1, E-selectin and the employed CD44 variants (Figure 1). P-selectin was the only significantly elevated cytokine after histamine application. Thus, in further experiments TNF-alpha was used for endothelial ICAM-1, VCAM-1, E-selectin and CD44 stimulation and histamine for P-selectin activation.

### RCC-endothelial cell interaction alters endothelial receptor expression

#### Evaluation for receptor surface expression

**Figure 2:**
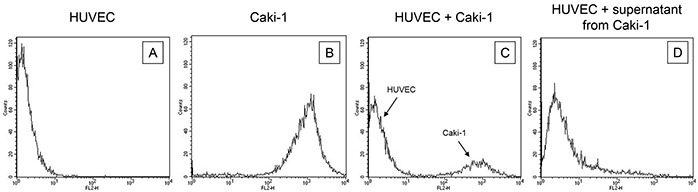
Purity of the HUVEC fraction was established by FACscan analysis, revealing **A.** a CM-Dil dye negative HUVEC and **B.** a CM-Dil dye positive Caki-1 fraction. Purity was also demonstrated for HUVEC in co-culture with **C.** CM-Dil dye marked Caki-1 and **D.** for HUVEC incubated with supernatant (culture medium) from Caki-1 cells. One representative of three separate experiments is shown.

Endothelial cells, HUVEC, HMEC-1 or HC-6014 were separated from co-culture with CM-Dil dye marked Caki-1 cells. Purity of the HUVEC fraction was shown by FACscan analysis, revealing a CM-Dil dye negative HUVEC and a CM-Dil dye positive Caki-1 fraction (Figure 2).

**Figure 3:**
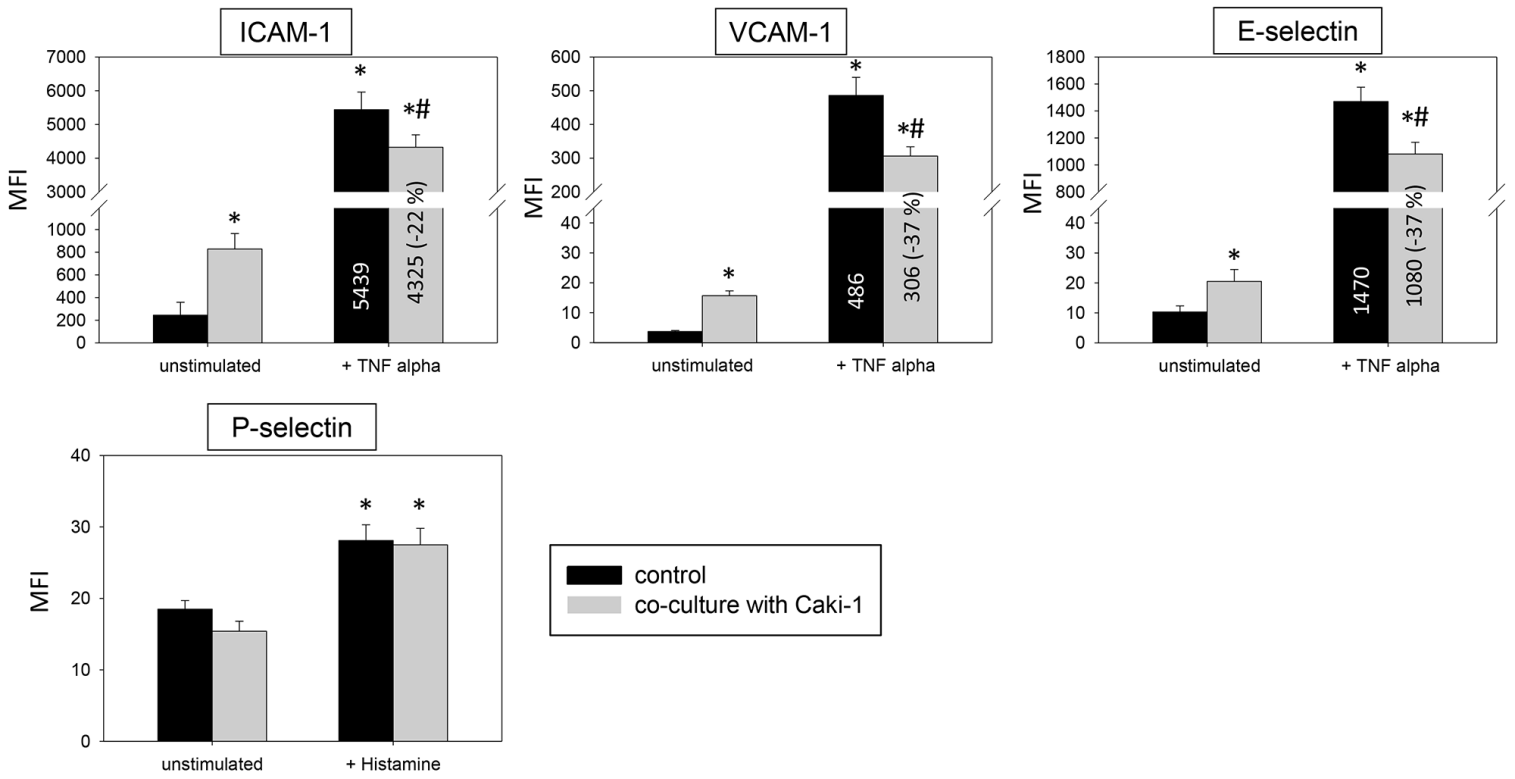
Endothelial surface expression of adhesion receptors in HUVEC after cytokine stimulation and/or co-cultivation with Caki-1 cells Expression of ICAM-1, VCAM-1 and E-selectin after 24h TNF-alpha [500 U/ml] and P-selectin after 10 min histamine [100 μM/ml] stimulation. MFI = mean relative fluorescence intensity. *indicates significant difference to untreated controls. #indicates significant difference to TNF-alpha/histamine stimulated HUVEC. n=5.

**Figure 4:**
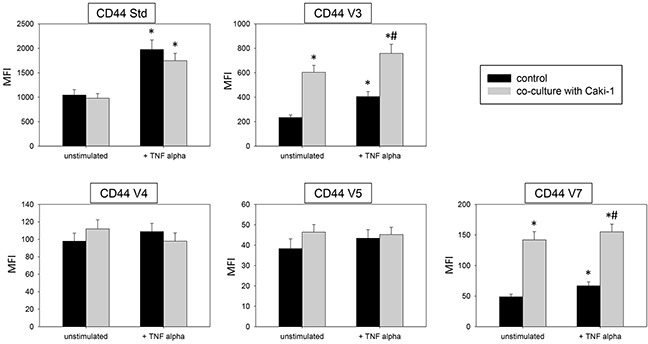
Endothelial surface expression in HUVEC of CD44 standard (Std), CD44 V3, CD44 V4, CD44 V5 and CD44 V7 after 24h TNF alpha [500 U/ml] stimulation and/or co-cultivation with Caki-1 cells MFI = mean relative fluorescence intensity. *indicates significant difference to controls. #indicates significant difference to TNF-alpha stimulated HUVEC. n=5.

Two models, a static and a flow based adhesion assay, were employed to evaluate receptor expression on conditioned endothelium. Under static conditions in un-stimulated HUVEC endothelial expression of the adhesion receptors ICAM-1, CD44 standard, CD44 V3, V4, V5 and V7 was low, while VCAM-1 and E-selectin expression was barely detectable. Adding Caki-1 to unstimulated HUVEC significantly enhanced the surface expression of endothelial ICAM-1, CD44 V3 and CD44 V7 on HUVEC, whereas VCAM-1, E-selectin, P-selectin, CD44 standard, CD44 V4 and CD44 V5 were not significantly affected (Figure 3 & 4). ICAM-1, VCAM-1, E-selectin, P-selectin, CD standard, CD44 V3 and CD44 V7 were significantly increased after TNF-alpha or histamine stimulation. Concomitant co-cultivation with Caki-1 resulted in significant inhibition of the receptor surface expression of ICAM-1, VCAM-1 and E-selectin (Figure 3), which was more pronounced when compared to Caki-1 co-cultivation. Notably, the TNF-alpha dependent elevation of CD44 V3 and CD44 V7 surface expression was significantly augmented in HUVEC when Caki-1 cells were present (Figure 4). Since endothelial P-selectin, CD44 standard, CD44 V4 and CD44 V5 were not affected by adding Caki-1 cells (Figure 3 & 4), these receptors were not included in subsequent studies.

**Figure 5:**
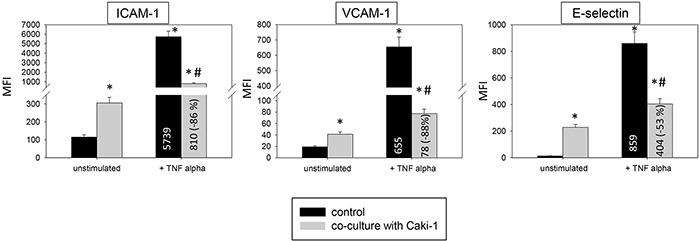
Endothelial surface expression of adhesion receptors on HMEC-1 and HC-6014 cells after TNF-alpha [500 U/ml] stimulation and/or co-cultivation with Caki-1 cells Expression of ICAM-1, VCAM-1 and E-selectin after 24h TNF-alpha [500 U/ml] stimulation. MFI = mean relative fluorescence intensity. *indicates significant difference to untreated controls. #indicates significant difference to TNF-alpha stimulated HUVEC. n=5.

The static adhesion assay was repeated using other endothelial cell lines. HMEC-1 or HC-6014 demonstrated similar alterations in receptor expression when conditioned with Caki-1 cells (Figure 5), compared to HUVEC. TNF-alpha evoked a significant elevation of ICAM-1, VCAM-1 and E-selectin expression. This effect was counteracted when Caki-1 cells were added to TNF-alpha activated endothelium.

**Figure 6:**
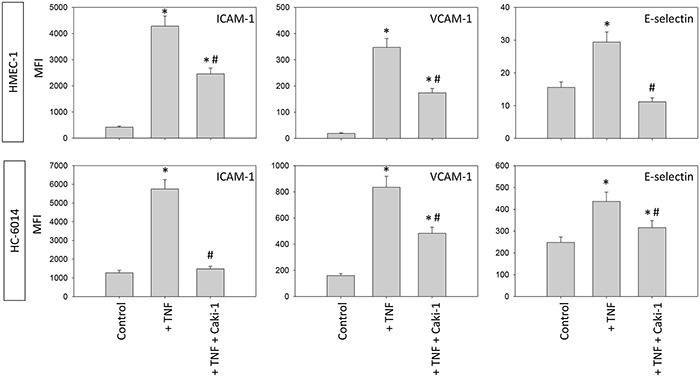
Endothelial surface expression of adhesion receptors on HUVEC after TNF-alpha [500 U/ml] stimulation and/or co-cultivation with Caki-1 cells under flow conditions Expression of ICAM-1, VCAM-1 and E-selectin after 24h TNF-alpha [500 U/ml] stimulation. MFI = mean relative fluorescence intensity. *indicates significant difference to untreated controls. #indicates significant difference to TNF-alpha/histamine stimulated HUVEC. n=6.

Under flow conditions similar modifications on HUVEC receptors were seen in the flow-based model (Figure 6). The surface expression of ICAM-1, VCAM-1 and E-selectin significantly increased in naïve HUVEC after adding Caki-1. Activating HUVEC with TNF-alpha led to a distinct increase of ICAM-1 along with VCAM-1 and E-selectin. In this experiment, all receptors were down-regulated following addition of Caki-1 cells. The endothelial response was even stronger than the response evoked in the static adhesion assay.

#### Evaluation of receptor protein level by Western blot

**Figure 7:**
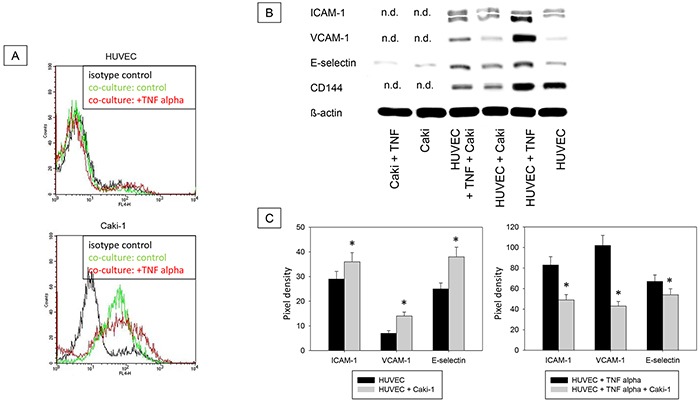
**A.** Magnet bead separation of Caki-1 and HUVEC from the co-culture by epithelial CD326. Black line: isotype control, green line: unstimulated cells, red line: TNF-alpha (24h, 500 U/ml) stimulated cells. One representative of three separate experiments is shown. **B.** Western Blot Analysis. Total protein content of ICAM-1, VCAM-1, E-selectin and CD144 (endothelial control) in Caki-1 and HUVEC cells separated by magnet beads. HUVEC + Caki = HUVEC separated from Caki-1 co-culture. Caki-1 and HUVEC cells, cultivated separately, served as controls. n.d. = not detectable. **C.** Pixel density of protein bands shown in B.

Magnetic beads were used to separate the epithelial CD326 receptor positive Caki-1 fraction and CD326 receptor negative HUVEC fraction (Figure 7A). Additionally, HUVEC purity was confirmed using the endothelial receptor CD144, which is not expressed on Caki-1 cells (Figure 7B). ICAM-1, VCAM-1 and E-selectin expression was low in unstimulated HUVEC but significantly rose upon co-cultivation with Caki-1 cells (Figure 7B & 7C). Adding TNF-alpha to HUVEC cells alone resulted in significantly increased endothelial receptor expression, as well, while co-culture with Caki-1 cells caused a significant decrease in ICAM-1, VCAM-1 and E-selectin in the HUVEC cells, compared to TNF-alpha stimulated HUVEC (Figure 7B & 7C). In Caki-1 cells ICAM-1 and VCAM-1 were not detectable and E-selectin was only marginally expressed (Figure 7B & 7C).

**Figure 8:**
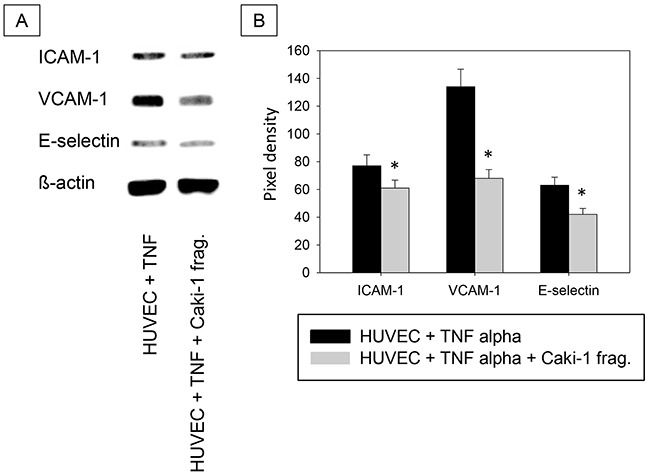
**A.** Western Blot Analysis. Total protein content of ICAM-1, VCAM-1 and E-selectin in HUVEC cells incubated with TNF-alpha (500 U/ml) and/or Caki-1 cell fragments for 24h. TNF = TNF-alpha, Caki-1 frag. = Caki-1 cell fragments. **B.** Pixel density of protein bands shown in A.

Addition of isolated Caki-1 cell fragments had similar effects on HUVEC (Figure 8), as those seen when Caki-1 cells were added to HUVEC. Figure 8 demonstrates down-regulation of ICAM-1, VCAM-1 and E-selectin when fragments were exposed to TNF-alpha stimulated HUVEC.

### Caki-1 conditioning of endothelium

To evaluate whether direct cell-cell contact or released soluble factors are responsible for endothelial cell conditioning, isolated Caki-1 cell fragments, membrane proteins or supernatants from Caki-1 culture or HUVEC-Caki-1 co-culture were added to HUVEC.

**Figure 9:**
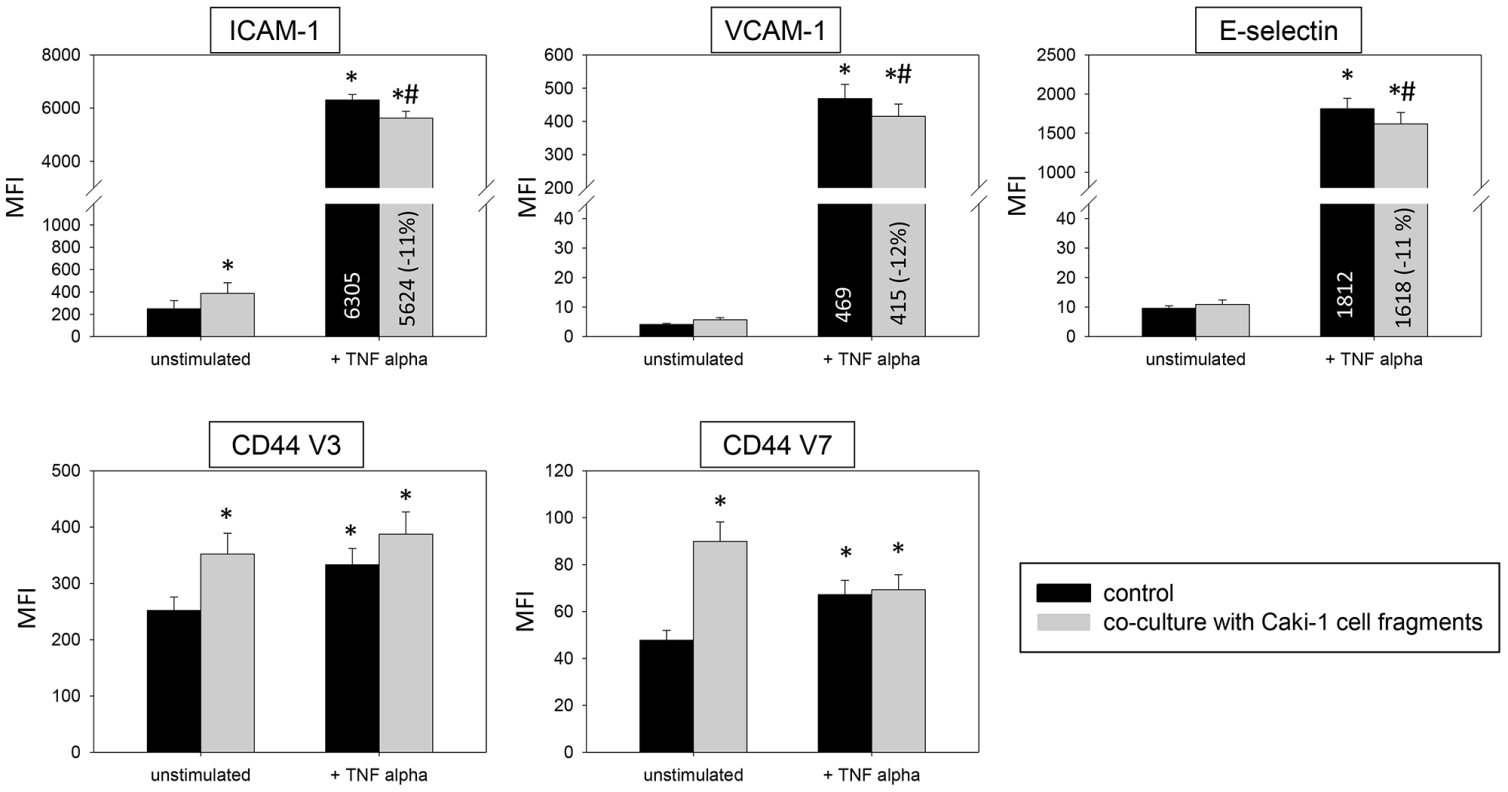
Impact of Caki-1 cell fragments on endothelial surface expression FACscan analysis of ICAM-1, VCAM-1, E-selectin, CD44 V3 and CD44 V7 after 12h TNF-alpha [500 U/ml] stimulation and/or co-cultivation with Caki-1 cell fragments. MFI = mean relative fluorescence intensity. *indicates significant difference to untreated controls. #indicates significant difference to TNF-alpha stimulated HUVEC. n=5.

**Figure 10:**
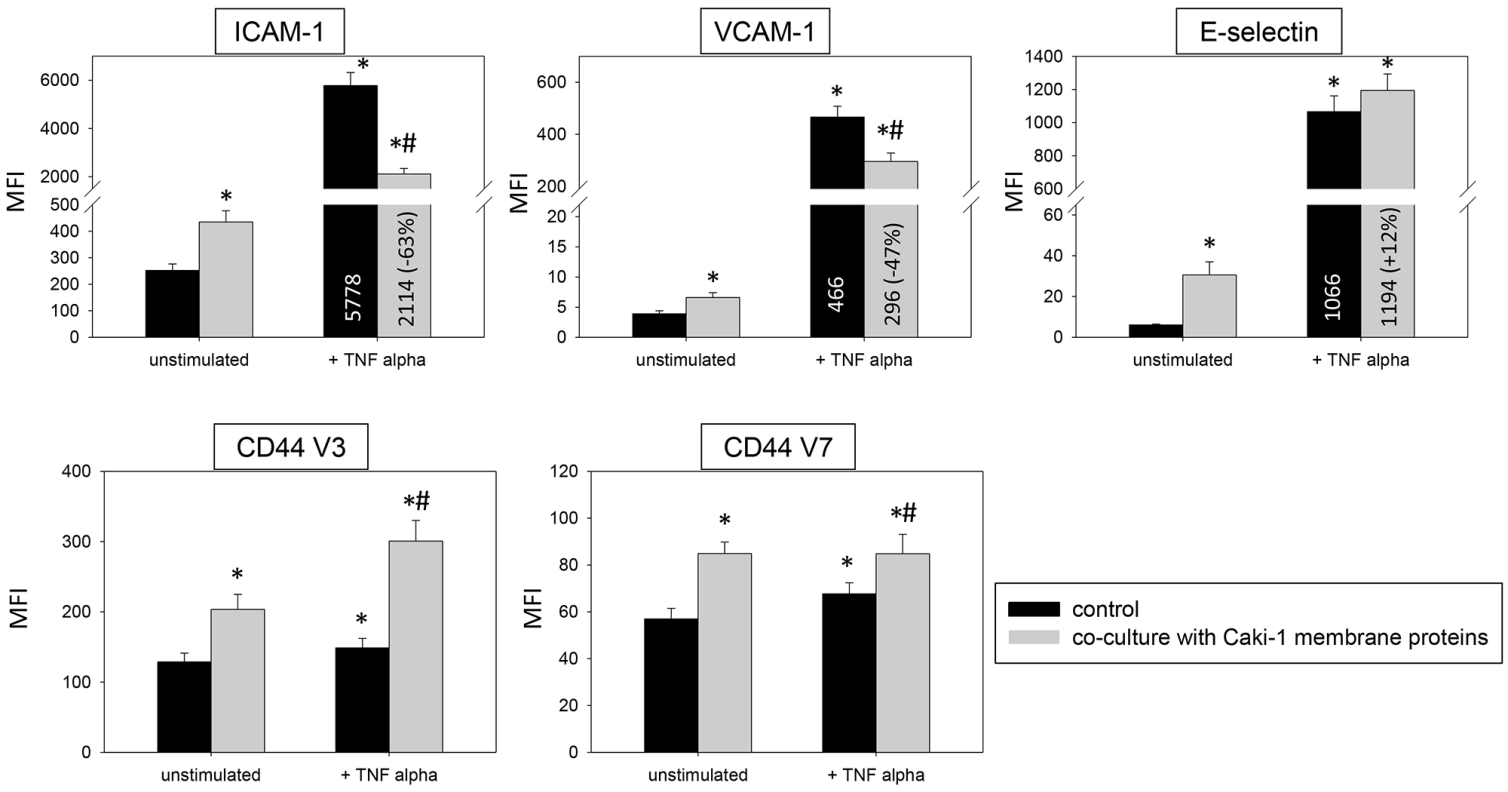
HUVEC conditioning of surface expression by Caki-1 membrane proteins FACscan analysis of ICAM-1, VCAM-1, E-selectin, CD44 V3 and CD44 V7 after 12h TNF alpha [500 U/ml] stimulation and/or co-cultivation with Caki-1 membrane proteins. MFI = mean relative fluorescence intensity. *indicates significant difference to untreated controls. #indicates significant difference to TNF-alpha stimulated HUVEC. n=5.

Introduction of Caki-1 cell fragments to unstimulated HUVEC contributed to significantly enhanced ICAM-1, CD44 V3 and CD44 V7 surface expression (Figure 9). Caki-1 cell fragments and TNF-alpha stimulation caused significantly diminished expression of ICAM-1, VCAM-1 and E-selectin on HUVEC cells, compared to TNF-alpha stimulation alone. The diminished expression with cell fragments and TNF-alpha (Figure 9) was less pronounced than that with Caki-1 co-culture and TNF-alpha (Figure 3 & 4). Furthermore, Caki-1 cell fragments together with TNF-alpha enhanced CD44 V3, but not CD44 V7 expression (Figure 9). Addition of isolated Caki-1 membrane proteins caused an increase in ICAM-1, VCAM-1, E-selectin, CD44 V3 and CD44 V7 on the surface of unstimulated HUVEC cells (Figure 10). In TNF-alpha and Caki-1 membrane protein treated HUVEC, ICAM-1 and VCAM-1, but not E-selectin expression was significantly reduced, compared to the TNF-alpha supplemented HUVEC. CD44 V3 and CD44 V7 became elevated after Caki-1 membrane proteins had been added to TNF-alpha stimulated HUVEC (Figure 10), similar to the elevation after co-culture with Caki-1 cells (Figure 4).

**Figure 11:**
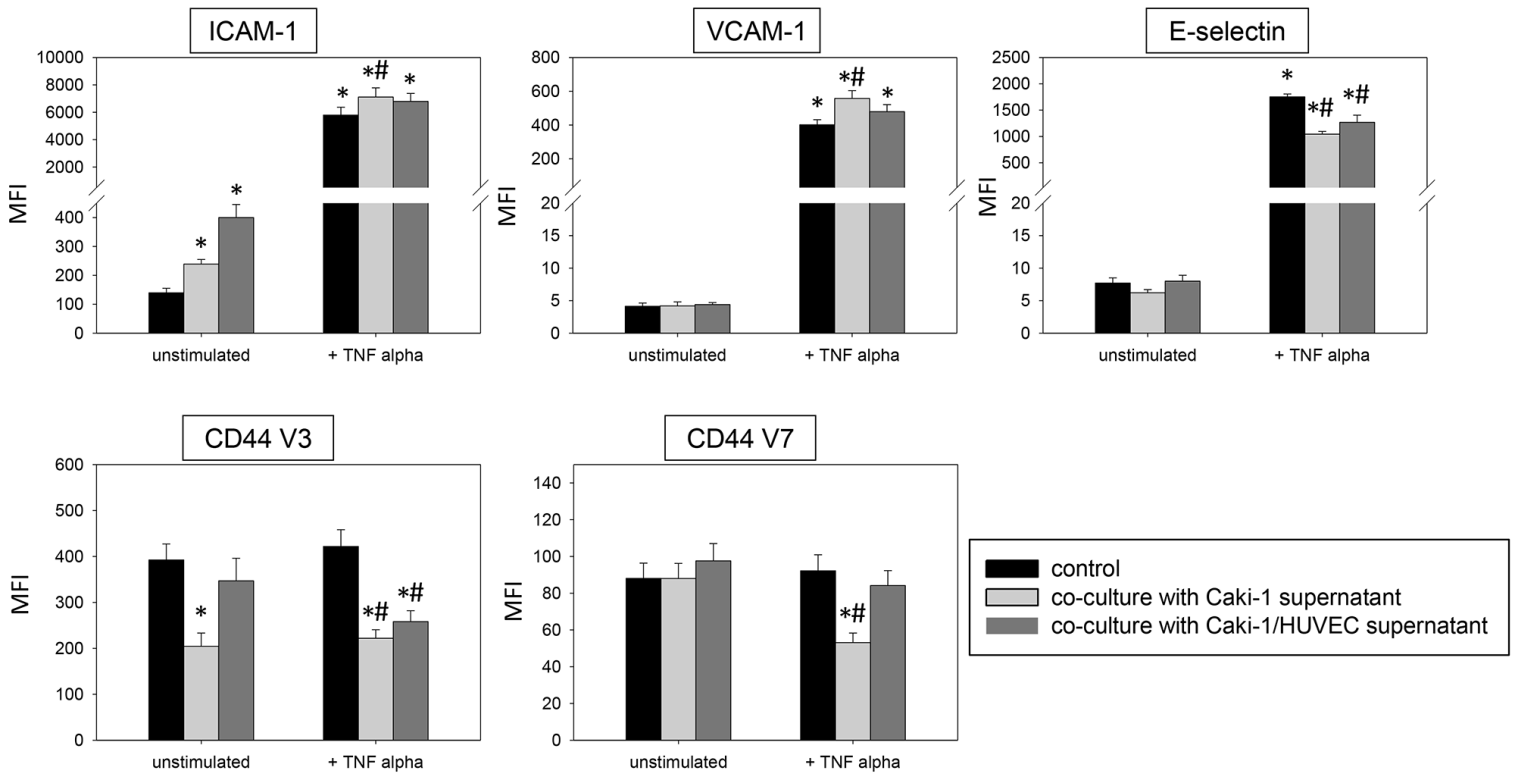
Influence of supernatants (culture medium) from Caki-1 or Caki-1-HUVEC co-culture FACscan analysis of ICAM-1, VCAM-1, E-selectin, CD44 V3 and CD44 V7 after 12h TNF-alpha [500 U/ml] stimulation and/or co-cultivation with Caki-1 or co-culture supernatants (culture medium). MFI = mean relative fluorescence intensity. *indicates significant difference to untreated controls. #indicates significant difference to TNF-alpha stimulated HUVEC. n=5.

In TNF-alpha stimulated HUVEC cells the supernatants of Caki-1 and HUVEC-Caki-1 co-culture significantly enhanced ICAM-1 and VCAM-1 and reduced CD44 V3 and CD44 V7 surface expression (Figure 11), as opposed to the effects on HUVEC found after Caki-1 cell contact (Figure 3 & 4). E-selectin expression was significantly diminished in TNF-alpha treated HUVEC after supernatant addition (Figure 11), reflecting the E-selectin decrease induced by Caki-1 cells (Figure 3). In general, effects were more pronounced in the presence of the Caki-1 supernatant, than with the co-culture supernatant.

### Relevance of endothelial receptors to leukocyte adhesion

**Figure 12:**
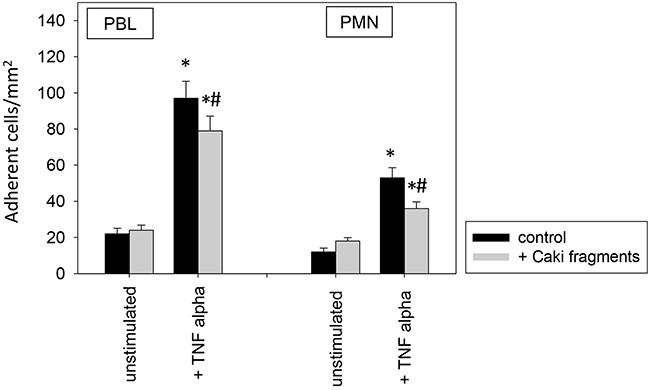
PBL and PMN adhesion to HUVEC after 12h pre-incubation with TNF-alpha [500 U/ml] and/or Caki-1 cell fragments Untreated HUVEC served as controls. Mean number of adherent PBLs and PMNs from five fields (0.25 mm^2^) were evaluated. *indicates significant difference to untreated controls. #indicates significant difference to TNF-alpha stimulated HUVEC. n=6.

**Figure 13:**
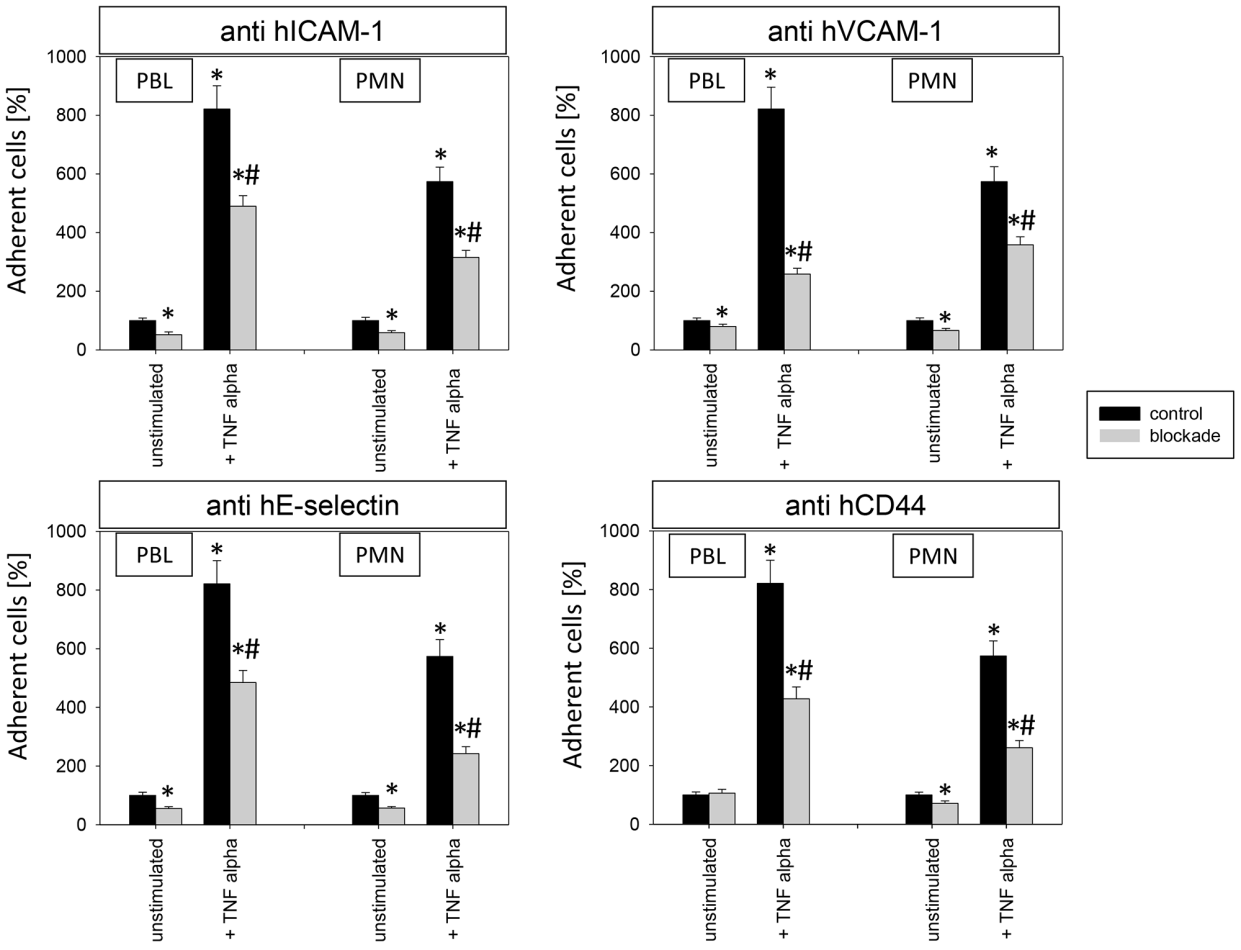
Impact of alteration in endothelial receptor expression on PBL and PMN adhesion to endothelium 12h stimulation with TNF-alpha [500 U/ml] and/or 1h functional blocking of endothelial ICAM-1, VCAM-1, E-selectin and CD44 by monoclonal antibodies. Unblocked HUVEC cells served as controls. Mean number of adherent PBLs and PMNs from five fields (0.25 mm^2^) were evaluated and expressed as % of adherent control cells (100%). *indicates significant difference to untreated controls. #indicates significant difference to TNF-alpha stimulated HUVEC. n=5.

To investigate relevance of receptor modification on leukocyte adhesion, PBL or PMN were added to HUVEC and adhesion was then evaluated (Figure 12). Adhesion of PBLs and PMNs to TNF-alpha activated endothelium significantly increased, compared to controls. This process was reversed when HUVEC were conditioned with Caki-1 cell fragments, as evidenced by a significant loss of PBL or PMN attachment (Figure 12). When functional blockade of endothelial ICAM-1, VCAM-1, E-selectin or CD44 was carried out, it was associated with reduced leukocyte binding to unstimulated endothelium and even less to TNF-alpha stimulated endothelium (Figure 13). Blocking VCAM-1 revealed stronger effects on adhesion of PBLs, whereas E-selectin and CD44 impairment resulted in stronger reduction of PMN (Figure 13).

## DISCUSSION

The impact of RCC cells on the expression of endothelial adhesion receptors and leukocyte attachment to endothelium was investigated.

RCC cells significantly modulated the endothelial receptor expression profile. Co-cultivation of unstimulated HUVEC with Caki-1 cells led to endothelial cell conditioning, evidenced by increased total and surface ICAM-1, CD44 V3 and CD44 V7. Previous findings demonstrate similar results and corroborate ICAM-1 impact on PBL and PMN adhesion to endothelium [10–12]. ICAM-1 is highly expressed in cancer [7] and involved in PBL and PMN adhesion to endothelium, as shown by function blocking studies. Furthermore, ICAM-1 regulates transendothelial leukocyte migration and endothelial permeability [7, 14, 15]. Thus, crosstalk between endothelium and RCC cells contribute to increased endothelial ICAM-1 expression and might enhance PBL and PMN binding to endothelium.

CD44 V4, V5 and V7 are relevant endothelial mediators in PMN-HUVEC interaction and crucial for PMN invasion [8]. Since endothelial CD44 V3 and V7 were up-regulated by Caki-1 cells, PMN binding to endothelium may increase, suggesting that enhanced endothelial receptor expression may contribute to leukocyte infiltration. Indeed, RCC is often characterized by immune cell infiltration [16]. However, these tumor infiltrating immune cells lose their immune competence [17], correlating with tumor growth [18], short disease-free survival [19] and adverse overall survival [20]. Furthermore, T cells that naturally infiltrate RCC are regulatory T cells (Tregs), which may suppress antitumor immune response [21]. They counterbalance the efficacy of effector T cells by interfering with local effector T cell response and correlate with poor prognosis.

RCC patients have significantly increased TNF-alpha serum levels [22]. To imitate the higher TNF-alpha serum level, HUVEC were stimulated by TNF-alpha in the present investigation. The applied concentration accords with other *in vitro* protocols, though it might be higher than TNF concentrations found in RCC tissue [23]. However, the present investigation was not aimed at evaluating the role of TNF-alpha per se, but rather to use TNF-alpha as a trigger to evoke maximum endothelial response. Indeed, TNF-alpha stimulation resulted in elevated endothelial surface ICAM-1, CD44 V3 and CD44 V7 expression and de novo synthesis of VCAM-1 and E-selectin, compared to unstimulated HUVEC. Surprisingly, adding Caki-1 to the TNF-alpha stimulated HUVEC evoked a significantly diminished endothelial ICAM-1, VCAM-1 and E-selectin expression, compared to HUVEC not influenced by Caki-1. Reduced endothelial ICAM-1, VCAM-1 and E-selectin was associated with decreased PBL and PMN adhesion, as shown by adhesion receptor blocking. Consistent with these findings, the correlation between TNF-alpha release and increased endothelial VCAM-1, ICAM-1, E-selectin and CD44 has previously been shown [8, 24, 25]. Expression of ICAM-1, VCAM-1 and E-selectin has been associated with endothelium-dependent leukocyte rolling [26], ICAM-1 and VCAM-1 especially for PMN rolling and firm PMN adhesion and migration [27]. All three receptors activate PMN and promote an inflammatory response [12]. Hence, compared to unstimulated HUVEC, RCC cells together with TNF-alpha appear to restrict leukocyte extravasation by reducing endothelial ICAM-1, VCAM-1 and E-selectin expression. This is in accordance with studies on colon carcinoma and melanoma, demonstrating significantly suppressed ICAM-1 and VCAM-1 expression, which have been shown to contribute to decreased leukocyte adhesion *in vivo* [28]. Furthermore, it has been shown that diminished endothelial E-selectin is accompanied by reduced leukocyte adhesion to activated endothelial cells [10]. In human squamous cell carcinomas and merkel cell carcinoma, inhibition of vascular E-selectin expression led to decreased leukocyte infiltration [29, 30]. Thus, in a TNF-alpha enriched environment, RCC cells may counteract immune recognition by decreasing endothelial ICAM-1, VCAM-1 and E-selectin expression to inhibit leukocyte extravasation.

In contrast to increased ICAM-1, VCAM-1 and E-selectin, endothelial CD44 V3 and V7 expression was enhanced in a TNF-alpha enriched environment with Caki-1 cells. This enhancement was also apparent without TNF-alpha enrichment. Less is known about the endothelial CD44 variants V3 and V7. Endothelial CD44 variants V4, V5 and V7 have been shown to be modulated after endothelial contact with neuroblastoma cells, altering PMN adhesion to endothelium [8]. Since Caki-1 cells contributed to up-regulation of ICAM-1, VCAM-1, E-selectin and CD44 V3 and V7 in unstimulated HUVEC, but down-regulation of ICAM-1, VCAM-1 and E-selectin in TNF-alpha stimulated HUVEC and further increased CD44 V3 and V7, conditioning of the endothelium by RCC could depend on the presence of TNF-alpha. TNF-alpha activation may contribute to a switch in leukocyte recruitment from primarily ICAM-1, VCAM-1 and E-selectin-dependent leukocyte binding in the unstimulated HUVEC towards CD44-mediated PBL and PMN adhesion to TNF-alpha stimulated HUVEC. This might lead to an aberrant composition of infiltrating leukocytes, favoring cytotoxically inactive cells, which could promote immune evasion. Whether the observed endothelial receptor expression actually leads to an immune suppressive tumor infiltrate is hypothetical, since PBL and PMN motility and cytotoxicity as well as PBL subtypes remain to be evaluated in further investigations.

Endothelial cell conditioning was induced by direct cell-cell contact between endothelium and tumor cell, as is the case with co-cultivation of HUVEC with RCC cell membranes. Caki-1 cell fragments and membrane proteins led to the same effects on adhesion receptor expression as co-culturing HUVEC with Caki-1 cells. However, supernatants from Caki-1 cell cultures had no such effects on HUVEC cells. Similar observations have been made for neuroblastoma, where endothelial receptors have also been shown to be altered by tumor-endothelial cell contact. Here, tumor supernatant had no effect on endothelium [8]. In the current study, E-selectin was the only exception. Endothelial E-selectin reduction, comparable to that of the Caki-1 co-culture model with HUVEC, was only detectable with supernatants. Notably, the impact of Caki-1 supernatant was stronger than with HUVEC-Caki-1 co-culture supernatant, indicating that a soluble factor released by Caki-1 takes place in the absence of HUVEC. In cancer associated endothelial cells of colon carcinoma and melanoma *in vivo*, TNF-alpha induced endothelial receptor up-regulation and leukocyte adhesion was abolished by factors released by the tumor [28, 31]. Thus, in RCC the direct (membrane) contact predominantly affects endothelial cell conditioning, whereas soluble factor release seems to be an additional feature for fine tuning, at least partially, via modulation of endothelial E-selectin.

Ongoing studies are in progress, dealing with phenotype and function of the particular leukocyte sub-populations, which attach to conditioned endothelium. It is still not clear whether down-regulation of the endothelial receptors analyzed in the present investigation correlate with a reduced invasion of leukocytes with tumor suppressive and cytotoxic properties. Particularly the neutrophil's role in tumor growth and progression is still a matter of debate. Although several investigations suggest a tumor-promoting function of PMN, proof of tumor-inhibitory actions have also been provided. Jensen et al. have observed a negative impact of intratumoral neutrophils in RCC patients [32], whereas neutrophils correlated negatively with tumor progression and survival in another study [33]. A subpopulation of activated neutrophils has been demonstrated to exert immunosuppression in RCC [34]. Since migration from the primary tumor across the basal membrane into the vasculature might be different from the process of metastatic settling, it would also be worthwhile to compare modification of endothelial receptor expression in primary versus secondary tumors.

This investigation shows that RCC cells significantly modulate the endothelial receptor expression profile and provides insight into the complex interaction between RCC cells, endothelium and immune cells. It is postulated that RCC cells condition endothelial cells by altering endothelial receptor expression, thereby regulating PBL and PMN influx into the tumor.

## MATERIALS AND METHODS

### Cell culture and cytokine stimulation

Renal carcinoma (RCC) cells Caki-1 were purchased from LGC Promochem (Wesel, Germany). The cells were grown and subcultured in RPMI 1640 medium (Seromed, Berlin, Germany) supplemented with 10% FCS, 20 mM HEPES-buffer, 100 IU/ml penicillin and 100 μg/ml streptomycin at 37°C in a humidified, 5% CO_2_ incubator. Subcultures from passages 5-24 were used.

Human umbilical vein endothelial cells (HUVEC) were harvested by enzymatic treatment with dispase (1 Unit/ml, Gibco/Invitrogen). They were cultured in Medium 199 (M199; Biozol, Munich, Germany), supplemented with 10% fetal calf serum (FCS), 10% pooled human serum, 20 μg/ml endothelial cell growth factor (Boehringer, Mannheim, Germany), 0.1% heparin, 100 ng/ml gentamycin and 20 mM HEPES-buffer (pH 7.4). Subcultures from passages 2-5 were selected for experimental use. The institutional ethics committee of the Goethe-University Hospital, Frankfurt, Germany, waived the need for consent, since HUVEC were used anonymously for *in vitro* assays with no link to patient data. To mimic a malignant environment where the immune system is stimulated, HUVEC were exposed to either TNF-alpha (500 U/ml, recombinant human TNF-a, PeProTech, Hamburg, Germany) for 12 - 24h, histamine (100 μM/ml, SIGMA-Aldrich, Taufkirchen, Germany) for 10 min, interleukin 1 alpha (500 U/ml, PeProTech, Hamburg, Germany) for 16h or prostaglandin E2 (10 μM/ml, SIGMA-Aldrich, Taufkirchen, Germany) for 10 min.

Immortalized human dermal microvascular endothelial cells (HMEC-1, kindly provided by the Department of Thoracic, Cardiac and Vascular Surgery, Goethe-University Hospital, Frankfurt am Main, Germany) were cultured in MCDB 131 Medium (w/o glutamine, Gibco/Invitrogen, Darmstadt, Germany), supplemented with 10% FCS, 5 μg/500 ml EGF, 100 IU/ml penicillin and 100 μg/ml streptomycin and 5 mM/500 ml glutamax at 37°C in a humidified, 5% CO_2_ incubator. Subcultures from passages 2-16 were selected for experimental use. Human kidney tumor-associated endothelial cells (HC-6014, PELOBiotech GmbH, Martinsried, Germany) were cultured in complete human endothelial cell medium (H1168-Kit, basal medium with growth factor supplement, PELOBiotech GmbH, Martinsried, Germany) at 37°C in a humidified, 5% CO_2_ incubator. Subcultures from passages 2-4 were selected for experimental use.

Peripheral blood lymphocytes (PBL) and polymorphonuclear neutrophils (PMN) were isolated from the venous blood of healthy adult volunteers by Polymorphprep (AXIS-SHIELD, Oslo, Norway), re-suspended in HUVEC medium and immediately used for experiments.

Cell viability was evaluated with trypan blue (Gibco, Darmstadt, Germany).

### Co-culture of endothelial (HUVEC) and tumor (RCC) cells

HUVEC cells were grown to sub-confluency in 25 or 75 cm^2^ culture flasks. 5 × 10^5^ Caki-1 cells were added to the HUVEC monolayer and the co-culture incubated for 24h at 37°C in a humidified, 5% CO_2_ incubator.

### Direct cell-cell contact versus soluble factors

To evaluate whether endothelial receptor expression was altered by direct tumor-endothelium contact or indirectly by solutes released into the cell culture supernatant (medium), HUVECs were treated with isolated Caki-1 cell fragments, membrane proteins or supernatants from Caki-1 culture or HUVEC-Caki-1 co-culture. Receptor expression was then evaluated.

For cell fragment isolation Caki-1 were detached and centrifuged at 1050 rpm at 4°C. The supernatant was decanted and the cell pellet shock frozen at −80°C for 15 min. Cell fragments were either immediately used for experiments or further stored at −80°C. To determine whether membrane proteins were responsible for membrane fragment effects, Caki-1 membrane proteins were isolated using the Mem-PER Plus Membrane Protein Extraction Kit (Thermo Scientific, Bonn, Germany), according to the manufactures' protocol. 100 μg/ml cell fragments or membrane proteins were added to HUVEC 12h prior to experiments.

Supernatant (medium) from Caki-1 culture or HUVEC-Caki-1 co-culture was collected after 24h incubation and added (1:2) to HUVEC 12h prior to experiments.

### Surface expression of endothelial adhesion receptors

#### Under static conditions

HUVEC, HMEC-1 or HC-6014 were separated from their co-culture with Caki-1 cells. For separation by flow cytometry Caki-1 cells were marked with the CellTracker CM-Dil dye (Mo Bi Tec, Göttingen, Germany) prior to co-culture (detectable by FACScalibur, BD Biosciences, Heidelberg; FL2-H channel histogram analysis; 1 × 10^4^ cells/scan). Cells were detached, washed in FACS buffer (PBS, 0.5% BSA) and incubated for 60 min at 4°C with monoclonal antibodies (mAbs) directed against the following endothelial adhesion receptors: ICAM-1 (CD54, HA58), VCAM-1 (CD106, 51-10C9), E-selectin (CD62E, 68-5H11), P-selectin (CD62P, AK-4, all: APC labelled, anti-human, mouse IgG1 *K*, 20 μl, BD Pharmingen, Heidelberg, Germany), CD44 standard (CD44std, SFF-2, mouse IgG1 *K*, 1:20) and CD44 variants V3 (VFF-327v3, 1:10), V4 (VFF-11, 1:20), V5 (VFF-8) and V7 (VFFF-9, 1:20, all: mouse IgG1, 20 μl, eBioscience, Frankfurt, Germany). All CD44 antibodies were labelled with APC (Lightning-Link Allophycocyanin – XL Conjugation Kit, Innova Biosciences, Cambridge, UK). APC mouse IgG1, K (MOPC-21, 20 μl, BD Pharmingen, Heidelberg, Germany) served as isotype control. Receptor expression of HUVEC was measured by flow cytometry using FACScalibur (BD Biosciences, Heidelberg; FL4-H channel histogram analysis; 1 × 10^4^ cells/scan). Data were expressed as *mean relative fluorescence intensity* (MFI).

#### Under flow conditions

HUVEC (3 × 10^5^ cells) were seeded onto fibronectin (25 μg/ml) pre-coated 6 cm^2^ plates (Falcon™ Easy-Grip Tissue Culture Dishes, Corning GmbH, Kaiserslautern, Germany) and incubated for 3 days as previously described. Confluent HUVEC were exposed to shear stress (3 dyn/cm^2^) for 24h with a cone-plate viscosimeter. Then, TNF-alpha (500 U/ml) and/or Caki-1 (CM-Dil stained) were added to HUVEC and the cells were further incubated under shear stress overnight. HUVEC separation and measurement of the surface expression was done as described under static conditions.

### Western blotting

For Western blot analysis Caki-1 were separated from co-culture with HUVEC by magnet bead separation with human CD326 MicroBeads (EpCam = epithelial cell adhesion molecule, 100 μl, Miltenyi Biotec, Bergisch Gladbach, Germany) using a MidiMACS Separator (Miltenyi Biotec, Bergisch Gladbach, Germany). FACscan analysis (FACScalibur, BD Biosciences, Heidelberg, Germany; FL4-H channel histogram analysis; 1 × 10^4^ cells/scan) with anti-human CD326 (EpCAM, 1B7, mouse IgG1 *K*, 10 μl, eBioscience, Frankfurt, Germany) as primary antibody was performed to exclude Caki-1 contamination in the HUVEC fraction. Allophycocyanin (APC) mouse IgG1 *K* (20 μl, BD Pharmingen, Heidelberg, Germany) served as secondary antibody and isotype control. To further verify purity, separation was proved by western blot analysis with an antibody against endothelial CD144 (anti-human CD144) and vascular endothelial (= VE)-cadherin (16B1, mouse IgG1, 1:250, eBioscience, Frankfurt, Germany).

To investigate the total content of endothelial receptors, HUVEC cell lysates were applied to a 7-12% polyacrylamide gel (depending on protein size) and electrophoresed for ~90 min at 100 V. The proteins were then transferred to nitrocellulose membranes. After blocking with non-fat dry milk for 1h, the membranes were incubated overnight with the following antibodies: ICAM-1 (anti-human CD54, HA58, mouse IgG1 *K*, 1:200, eBioscience, Frankfurt, Germany), VCAM-1 (CD106, 6G9, mouse IgG1, 1:500, EMELCA Bioscience, Breda, NL), E-selectin (CD62E, rabbit, 1:1000, Abbiotec, San Diego, CA, US) and CD144 (VE-Cadherin, 16B1, mouse IgG1, 1:250, eBioscience, Frankfurt, Germany). HRP-conjugated goat anti-mouse IgG and goat anti-rabbit IgG (both: 1:5.000; Upstate Biotechnology, Lake Placid, NY, US) served as secondary antibodies. The membranes were briefly incubated with ECL detection reagent (ECL™, Amersham/GE Healthcare, München, Germany) to visualize the proteins and then analyzed by the Fusion FX7 system (Peqlab, Erlangen, Germany). β-actin (1:1000; Sigma, Taufenkirchen, Germany) served as internal control. No adequate antibodies for CD44 variants were available, so that these receptors could not be evaluated. The same was performed with HUVEC, pre-incubated for 24h with TNF-alpha and/or Caki-1 cell fragments. Gimp 2.8 software was used to perform pixel density analysis of the protein bands by calculating the ratio of protein intensity × 100/β-actin intensity.

### HUVEC-immune cell interaction

To analyze leukocyte-HUVEC interaction, HUVEC were transferred to 6-well multiplates (Falcon Primaria; BD Biosciences) in complete HUVEC-medium. When confluency was reached, 5 × 10^5^ PBL or PMN were then added to the HUVEC monolayer for 1h. Subsequently, non-adherent tumor cells were washed off using warmed (37°C) Medium 199. The remaining cells were fixed with 1% glutaraldehyde. Adherent tumor cells were counted in five different fields of a defined size (5 × 0.25 mm^2^), using a phase contrast microscope, and the mean cellular adhesion rate was calculated. To determine whether the altered endothelial receptors influence immune cell adhesion to HUVEC, functional receptor blockade was performed. HUVEC were incubated for 60 min with 10 μg/ml function blocking monoclonal antibodies (mAbs) directed against ICAM-1 (CD54, BBIG-I1), VCAM-1 (CD106, BBIG-V1), E-selectin (CD62E, BBIG-E1, all: mouse IgG1, R&D Systems, Wiesbaden-Nordenstadt, Germany) and CD44 (5F12, LifeSpan BioSciences, Eching, Germany) and the leukocyte adhesion experiment was then carried out.

### Statistics

All experiments were performed 3-6 times. Statistical significance was determined by the Wilcoxon-Mann-Whitney-U-test. Differences were considered statistically significant at a p-value less than 0.05.
