# Correction: Renal cell carcinoma alters endothelial receptor expression responsible for leukocyte adhesion

**DOI:** 10.18632/oncotarget.14438

**Published:** 2017-01-02

**Authors:** Eva Juengel, Geraldine Krueger, Jochen Rutz, Karen Nelson, Isabella Werner, Borna Relja, Barbara Seliger, Beate Fisslthaler, Ingrid Fleming, Igor Tsaur, Axel Haferkamp, Roman A. Blaheta

**Present**: Due to an error in the production process, figures 5 and 6 were switched. Each figure legend is correct, but is associated with the wrong figure.

Corrected: The proper figure legends are provided below. The publisher sincerely apologizes for this oversight.

Original article: Oncotarget. 2016; 7(15):20410-24. doi: 10.18632/oncotarget.7804.

**Figure 5:**
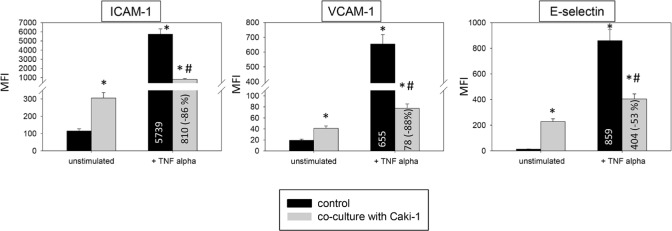
Endothelial surface expression of adhesion receptors on HUVEC after TNF-alpha [500 U/ml] stimulation and/or co-cultivation with Caki-1 cells under flow conditions Expression of ICAM-1, VCAM-1 and E-selectin after 24h TNF-alpha [500 U/ml] stimulation. MFI = mean relative fluorescence intensity. *indicates significant difference to untreated controls. #indicates significant difference to TNF-alpha/histamine stimulated HUVEC. n=6.

**Figure 6:**
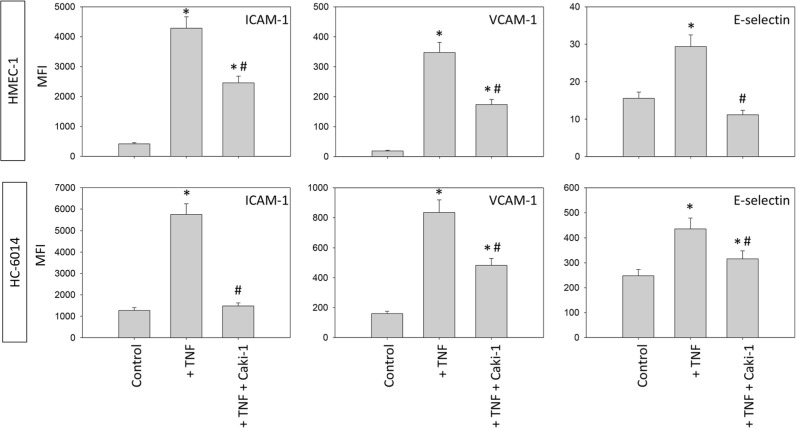
Endothelial surface expression of adhesion receptors on HMEC-1 and HC-6014 cells after TNF-alpha [500 U/ml] stimulation and/or co-cultivation with Caki-1 cells Expression of ICAM-1, VCAM-1 and E-selectin after 24h TNF-alpha [500 U/ml] stimulation. MFI = mean relative fluorescence intensity. *indicates significant difference to untreated controls. #indicates significant difference to TNF-alpha stimulated HUVEC. n=5.

